# Outcomes following transcatheter repair in patients with functional mitral regurgitation not receiving guideline directed medical therapy in Israel

**DOI:** 10.1186/s12872-023-03344-2

**Published:** 2023-06-16

**Authors:** David Leibowitz, Dan Haberman, Sorel Goland, Jacob George, Ronen Beeri, David Planer, Rafael Wolf, Becky Kutsher, Tal Hasin, Mony Shuvy

**Affiliations:** 1 Department of Cardiology, Faculty of Medicine Hadassah, Hebrew University Medical Center, Jerusalem, Israel; 2 Department of Cardiology, Kaplan Medical Center, Rehovot, Israel; 3 Department of Cardiology, Shaare Zedek Medical Center, Jerusalem, Israel; 4 Coronary Care Unit, Hadassah Medical Center Mount Scopus Jerusalem, Jerusalem, Israel

**Keywords:** Mitral regurgitation, Transcatheter repair, Congestive heart failure

## Abstract

**Background:**

Transcatheter edge to edge repair (TEER) improves prognosis in patients with functional mitral regurgitation (FMR) receiving guideline directed medical therapy (GDMT). Many patients with FMR do not receive GDMT and the utility of TEER in this population remains unclear.

**Methods:**

We retrospectively studied patients undergoing TEER. Clinical, echocardiographic and procedural variables were recorded. GDMT was defined as use of RAAS inhibitors and MRAs unless GFR was under 30 as well as beta blockers. The primary endpoint of the study was one year mortality.

**Results:**

168 patients (mean age 71.3 ± 9.3; 66% males) with FMR who underwent TEER were included of whom 116 (69%) received GDMT at the time of TEER and 52 (31%) did not. There were no significant demographic or clinical differences between the groups. There were no significant differences in procedural success and complications between groups. One year mortality was identical in the two groups (15% vs. 15%; RR 1.06, CI 0.43–2.63, P = 0.90).

**Conclusions:**

Our findings suggest that procedural success and one year mortality following TEER was not significantly different in HFREF patients with FMR with or without GDMT. Larger, prospective studies are necessary to define the benefit of TEER in this population.

## Background

Functional mitral regurgitation (FMR) is a common clinical finding in patients with congestive heart failure and reduced ejection fraction (HFREF) [1]. The presence of FMR is associated with increased morbidity and decreased survival in such patients [2]. Randomized trials have demonstrated improved prognosis following transcatheter edge to edge repair (TEER) implantation in patients with FMR and HFREF receiving guideline directed medical therapy (GDMT) [3]. Recent European Society of Cardiology guidelines for the management of valvular disease give TEER a IIa recommendation in symptomatic patients with FMR receiving GDMT [4]. However, even in the pivotal clinical trials utilizing TEER many patients did not receive full GDMT [3]. HFREF patients with FMR do not receive GDMT for a variety of reasons. Registry data suggest at least 30% of HFREF patients do not receive GDMT and that this subgroup has a significantly higher mortality than patients receiving GDMT [5, 6]. Underutilization of GDMT persists even after one year follow up of HFREF patients [7]. While issues related to socioeconomic factors and access to health care partially explain this phenomenon, poor tolerance of GDMT is also an important factor [8]. The utility of TEER in this important patient population remains unclear. The results of medical interventions may be different in different countries as well as differing ethnic and socio-economic groups. [9, 10]. The objective of this study was to examine the impact of GDMT on one year outcomes following TEER in a broad patient population in Israel.

## Methods

We retrospectively studied a cohort of patients undergoing TEER in three Israeli medical centers; Hadassah Hebrew University Medical Center, Shaare Zedek Medical Center and Kaplan Medical Center between January 2014 and December 2020. Demographic, clinical, echocardiographic and procedural variables were recorded for all patients whose participation in the database was approved by the institutional review board of both institutions. All patients underwent routine transthoracic echocardiography with assessment of ejection fraction (EF) as part of their assessment prior to TEER. Patients with severe FMR and EF ≤ 40% were eligible for inclusion in the study. Patients who underwent emergent TEER for acute FMR were excluded from the study. Procedural success was defined as moderate or less residual MR with no in hospital mortality or emergent conversion to surgery. GDMT was defined as the use of beta blockers, renin-angiotensin-aldosterone system (RAAS) inhibitors and mineralocorticoid receptor antagonists (MRAs) unless glomerular filtration rate was under 30. Patients were generally followed up in CHF clinic every 3 months with assessment of NYHA class at intervals ranging from 6 to 12 months. GDMT was defined as use of RAAS inhibitors and MRAs unless GFR was under 30 as well as beta blockers. The primary endpoint of the study was one year mortality which was assessed via the institutional and Ministry of Health databases.

### Statistical analysis

Categorical variables are reported as n (%). The association between categorical variables was assessed using the Chi-square test or Fishers exact test as appropriate. Continuous variables are reported according to their distribution. Normally distributed variables are reported as mean ± standard deviation and compared using the t-test. Non normally distributed variables are reported as median and interquartile range and compared using Mann-Whitney U test. The variables which were found to be significantly associated with mortality using the univariate approach, were entered into a multivariate logistic regression model with backwards selection for a dichotomous dependent variable (mortality yes/no). The Kaplan-Meier model was applied for assessing the effect of GDMT on survival, with the log rank test for the comparison of survival curves. All statistical tests applied were two-tailed, and a p-value of 0.05 or less was considered statistically significant. All statistical analysis was done using SPSS software.

## Results

**Fig. 1:**
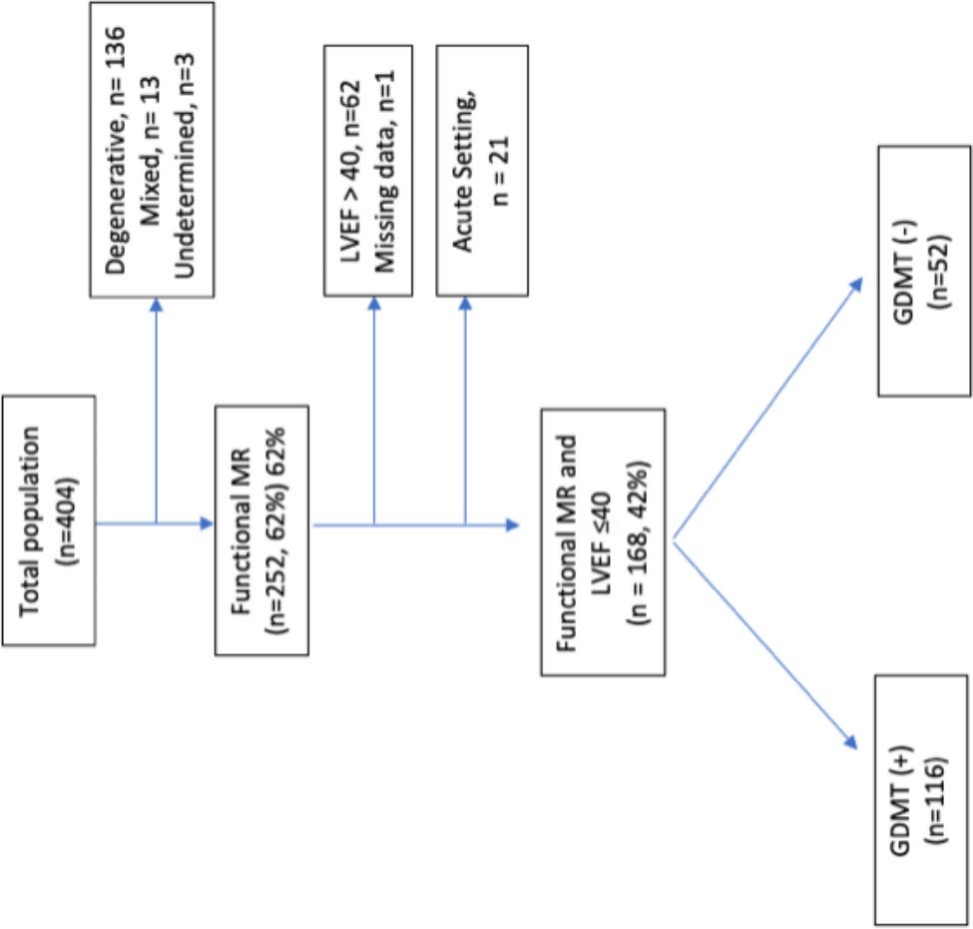
Study flow chart Abbreviations: GDMT, guideline directed medical treatment; LVEF; left ventricle ejection fraction; MR, mitral regurgitation

Of the 404 patients enrolled in the institutional databases, 168 patients (mean age 71.3 ± 9.3; 66% males) with FMR who underwent TEER and had an EF < 40% were included in the study. (Fig. 1) The mean follow-up time was 308 ± 102 days (median 365 days). One-hundred-sixteen patients (69%) were treated according to guideline directed medical treatment (GDMT +), and the other 52 patients (31%) were not treated according to GDMT (GDMT-).

Baseline clinical characteristics of study population before TEER are shown in Table 1. There is a male predominance in both groups with a higher prevalence of male gender (77% vs. 61%, P = 0.05) in the GDMT- group. There were no significant differences in background illnesses between the two groups. Not surprisingly, there was a significant difference in medical treatment. Patients who were not fully treated with GDMT had lower rate of treatment with: ACE-I/ARBs (63% vs. 84%, p < 0.01), beta blockers (79% vs. 99%, p < 0.01) and MRAs (17% vs. 62%, p < 0.01) compared with GDMT + patients. However, there was no significant difference in furosemide treatment (94% vs. 93%, p = 0.76) or furosemide mean dose (64 mg vs. 74 mg, p = 0.33). The number of CRT implantations was low and similar in both groups. No significant differences were observed in baseline echocardiographic and hemodynamic characteristics.

**Table 1:** – Baseline characteristics

Clinical characteristics	Patient population			P-value
	**168**	**GDMT (-), n = 52**	**GDMT (+), n = 116**	
**Age, Mean years**	71.3 ± 9.3	71.2 ± 9.1	71.3 ± 10.3	0.95
Male, %	111 (66)	40 (77)	71 (61)	0.05
BMI, Mean	26.9 ± 4.8	26.3 ± 5.1	27.2 ± 4.7	0.64
Hypertension, %	143 (86)	45 (88)	98 (85)	0.60
Diabetes Mellitus, %	69 (42)	21 (42)	48 (43)	0.96
Hyperlipidemia, %	132 (80)	40 (78)	92 (80)	0.82
Past MI, %	86 (51)	29 (56)	57 (49)	0.54
Past CABG %	63 (37)	20 (39)	43 (37)	0.81
EUROSCORE, Mean %	11.5 ± 11.4	10.9 ± 11.3	11.8 ± 11.6	0.47
Atrial Fibrillation, %	79 (47)	29 (55)	50 (43)	0.19
HF-related hospitalizations (During last year), %	144 (86)	42 (81)	102 (88)	0.24
Hgb, g/dL	11.6 ± 1.7	11.8 ± 1.7	11.5 ± 1.7	0.49
GFR, ml/min	54.1 ± 28.2	55.7 ± 28.9	53 ± 28.2	0.73
Mean MR grade	3.7 ± 0.51	3.72 ± 0.51	3.72 ± 0.51	0.91
LVEF, %	29.3 ± 6.9	30 ± 5.9	29 ± 7.3	0.58
LV dysfunction grade, Mean	3.17 ± 0.76	3.13 ± 0.59	3.19 ± 0.83	0.332
LVEDD, mm	62.3 ± 8.6	61.8 ± 7.1	62.55 ± 9.1	0.98
sPAP, mmHg	53.1 ± 14.3	53 ± 13.5	53.1 ± 13.5	0.84
LAP V-Wave, mmHg	33 ± 12.8	31.7 ± 8.2	33.9 ± 15.3	0.58
Base NYHA, grade	3.3 ± 0.54	3.3 ± 0.5	3.4 ± 0.6	0.23
**Cardiac-related drug therapy**:				
ACE-inhibitors or ARBs,%	131 (78)	33 (63)	98 (84)	**< 0.01**
Beta blockers,%	156 (93)	41 (79)	115 (99)	**< 0.01**
MRA,%	81 (48)	9 (17)	72 (62)	**< 0.01**
Furosemide,%	157 (94)	49 (94)	108 (93)	0.76
Fusid Dose, Mg	70.46 ± 47.2	64.4 ± 65.6	73.4 ± 48	0.33
ICD	24 (14)	6 (12)	18 (16)	0.44
CRT	13 (8)	4 (6)	9 (8)	0.67

Abbreviations: BMI, body mass index; CABG, coronary artery bypass grafting; CKD, chronic kidney disease; CRT, cardiac resynchronization therapy; EuroSCORE II, European System for Cardiac Operative Risk Evaluation; ICD, implantable cardioverter defibrillator; GFR, glomerular filtration rate; LAP, left atrial pressure; LVEF, left ventricular ejection fraction; MR, mitral regurgitation; NYHA, New York Heart Association functional class; sPAP, systolic pulmonary artery pressure

**Fig. 2:**
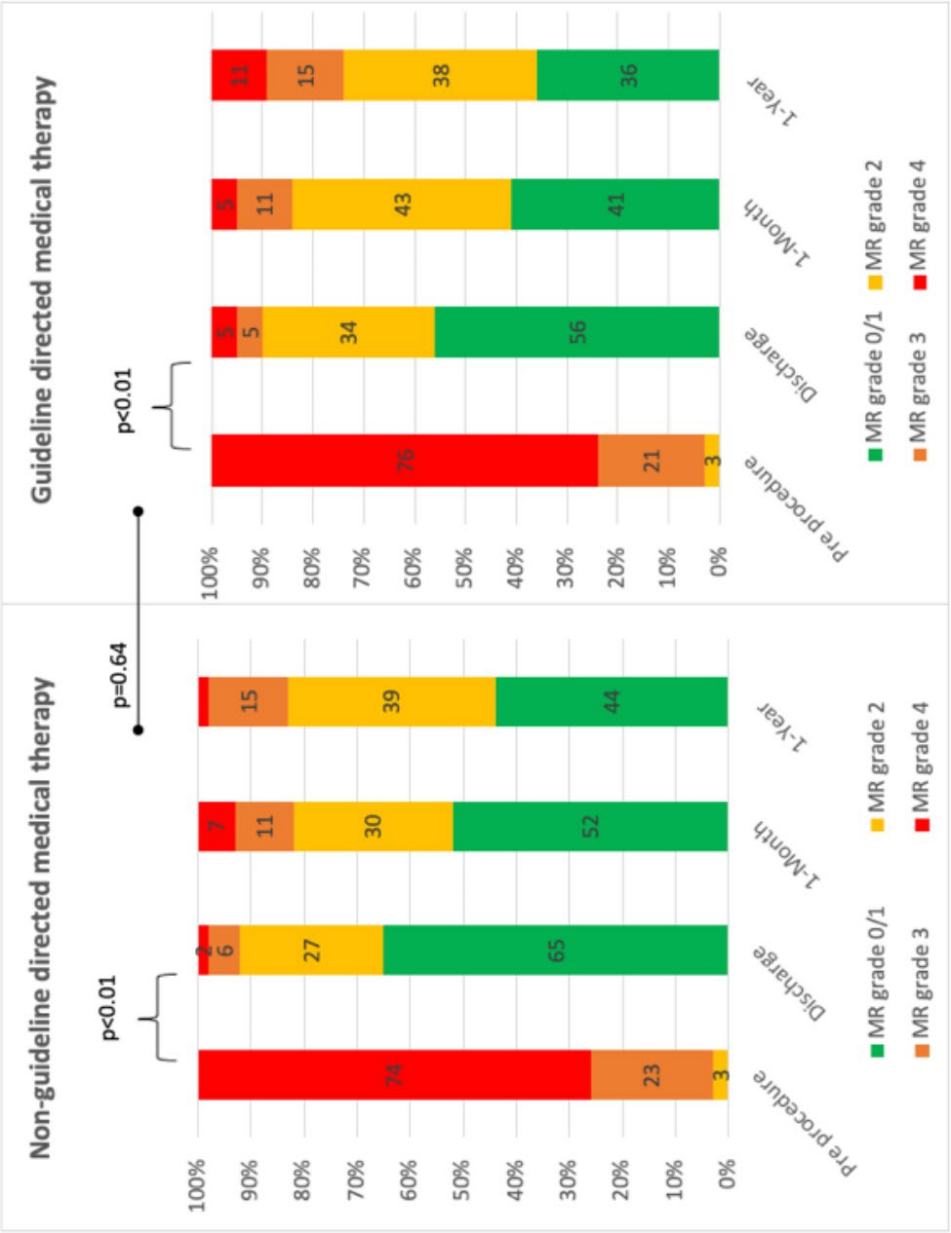
– Mitral regurgitation grade before the procedure, at discharge, 1-month and 1-year follow up

**Fig. 3:**
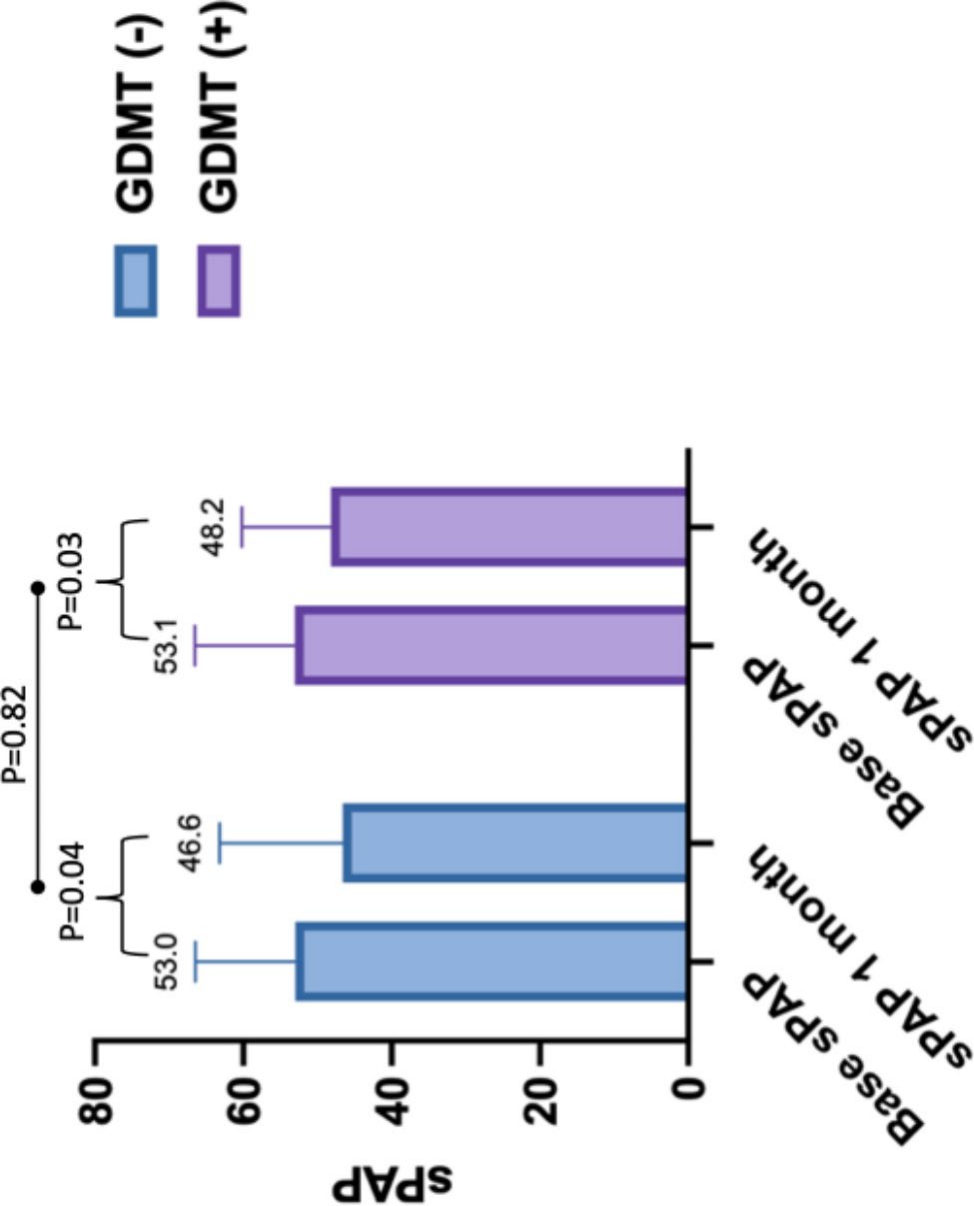
– Changes in estimated pulmonary artery pressure

**Fig. 4:**
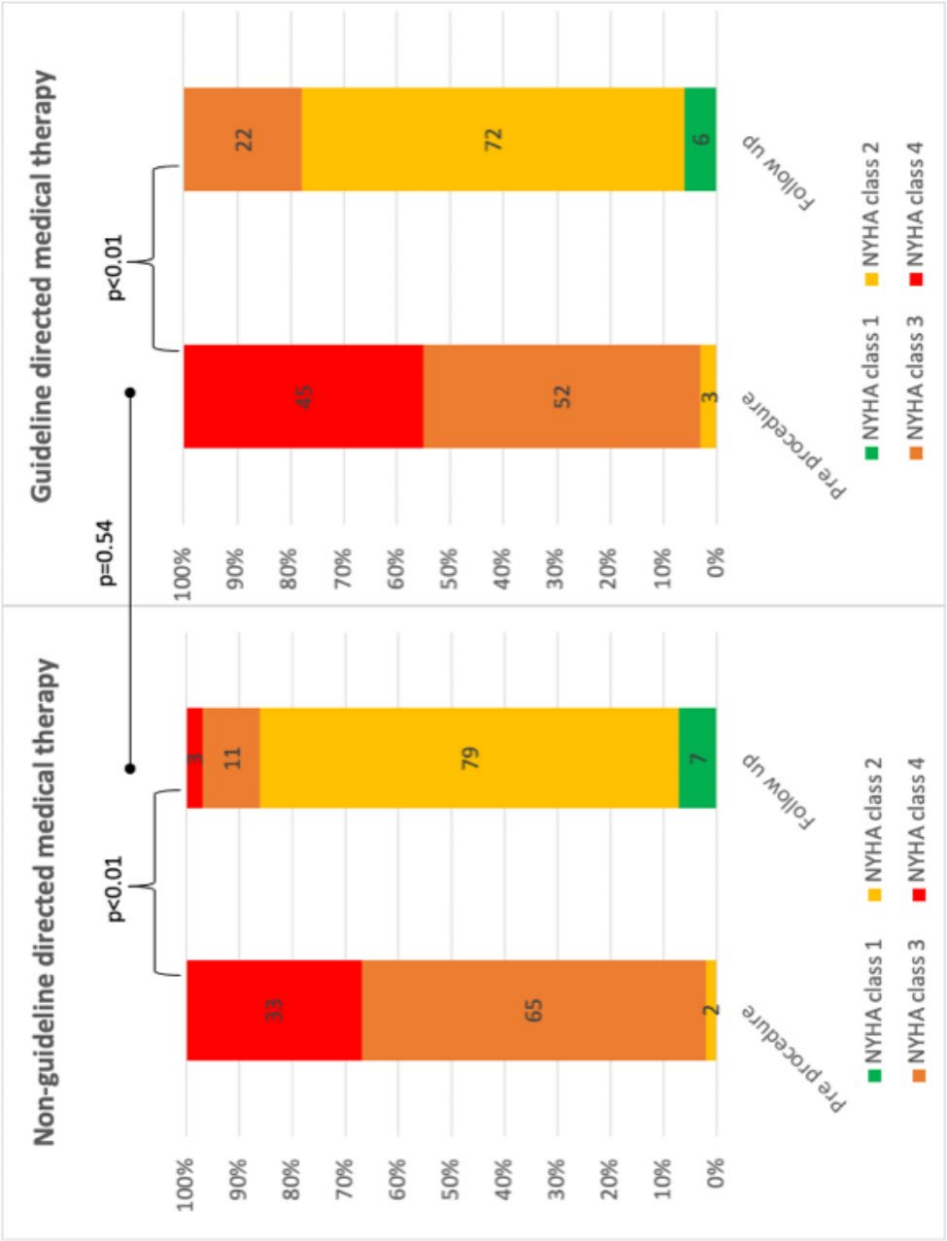
Changes in New York Heart Association class

Procedural success was high in both the GDMT- and GDMT + groups (92% vs. 89%, P = 0.78) and major complication rare was low (1.9% vs. 2.6%, p = 0.34). (Table 2) There was no difference in mean number of clips implanted (1.6 ± 0.6 vs. 1.8 ± 0.6, P = 0.66) or mean MR grade reduction before and after the procedure (2.2 ± 0.11 vs. 2.2 ± 0.1, P = 0.64). (Fig. 2). There were significant improvements in NYHA class and estimated pulmonary artery systolic pressure within both the GDMT + and the GDMT – groups without differences between the two groups. (Figures 3 and 4)

**Table 2:** – Procedural and clinical outcomes

Outcomes	GDMT (-), n = 52	GDMT (+), n = 116	P-value
Procedural success, %	92	89	0.78
Major complication, % (n)	1.9 (1)	2.6 (3)	0.98
Clips implanted, mean	1.6 ± 0.6	1.8 ± 0.6	0.66
MR reduction, Mean grade	2.23 ± 0.12	2.2 ± 0.1	0.64
Mitral valve gradient post (mmHg)	2.3 ± 3.4	2.1 ± 4.2	0.78
In-hospital mortality, %	1.9	0.9	0.52
6-Month mortality, %	10.3	7.4	0.63
1-Year mortality, %	15.4	14.7	0.90
I year rehospitalizations %	33.3	12.1	0.26

Abbreviations: MR, mitral regurgitation

**Fig. 5:**
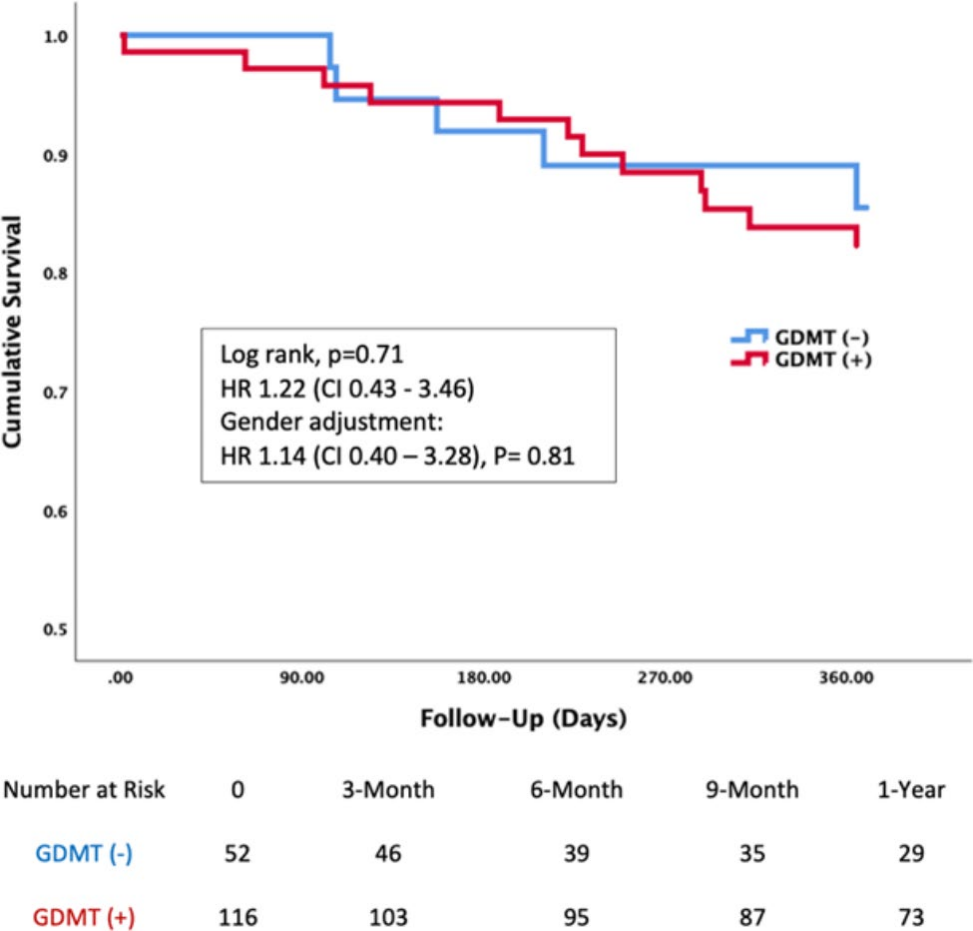
– Kaplan-Meier 1-year survival curve for patients treat with and without GDMT Abbreviations: CI, confidence interval; GDMT, guideline directed medical treatment, HR, hazard ratio

There was one case in each group of in-hospital mortality. One-year mortality was 15% in both groups. Kaplan-Meier curves for 1-year survival are shown in Fig. 5. No difference in mortality at one year is observed between the two groups (hazard ratio 1.22, confidence interval 0.43–3.46, p = 0.71). These results were unchanged after adjustment for the significant difference in male gender between the two groups (hazard ratio 1.14, confidence interval 0.40–3.28, p = 0.81).

## Discussion

Transcatheter mitral valve edge-to-edge repair (TEER) is an alternative to mitral valve surgery in patients with severe MR and high surgical risk. TEER is recommended in patients with chronic HF and severe FMR who remain symptomatic while treated with GDMT(4). However, in the real world many HFREF patients are not treated with GDMT due to various reasons such as comorbidities and side effects. In the current analysis, we found that almost half of patients treated with TEER did not receive GDMT. In this population, TEER procedure was safe and effective; MR was decreased, hemodynamic improvement was observed and one-year outcomes were similar to the rest of the cohort.

Treatment with GDMT is highly important in HFREF patients and it is associated with improved QOL and reduced mortality [11, 12]. The current guidelines highlight the importance of implementation of GDMT, as well as the titration of GDMT to doses achieved in randomized clinical trials [4]. Management of patients with FMR in HF is particularly challenging. Mortality is increased in patients with HF with FMR, and is related directly with increasing severity of the degree of FMR [13]. Management of patients with HF and severe FMR consists of optimizing medical therapy as first line therapy. Furthermore GDMT and cardiac resynchronization therapy may improve FMR and preclude the need for an invasive TEER procedure [14, 15].

Unfortunately, the multidrug regimen of GDMT is difficult to implement and optimize in a real world population. Data from more than 100,000 in the “Optum Integrated File” suggest that only 17.8% were treated with GDMT [5]. The reasons that HFREF patients were not optimally managed on GDMT include lack of guidelines implementation, drug tolerability, as well as patient comorbidities and preferences [16]. The CHAMP-HF (Change the Management of Patients with Heart Failure) registry included chronic HFREF patients receiving at least 1 oral medication for management of HF. Among eligible patients, only 1% were simultaneously receiving target doses of ACE/ARB/ARNI, beta-blocker, and MRA. Older patients, those with comorbidities, lower blood pressure, lower functional class, renal insufficiency, and recent HF hospitalization all had lower utilization of GDMT [6]. The outcomes of patients not on GDMT are worse although this may be because the inability to tolerate GDMT itself might be related to worse outcomes. Similarly, data from the 194 hospitals participating in Get With The Guidelines (GWTG)–Heart Failure program suggest that the use of GDMT is lower in patients at higher risk of mortality both because of higher rates of contraindications to therapy and lower rates of use among eligible patients [17]. The “risk-treatment paradox” where the HF patients at greatest need, such as those with advanced HF and severe MR, were less likely to receive appropriate medical therapy suggests that additional therapies should be considered [18].

The management of patients with severe FMR who do not receive GDMT is undetermined and it is unclear if TEER should be offered to this population. This is an important clinical question. For example in the COAPT study actual utilization rate of GDMT with only 68.5% of participants receiving RAAS inhibition. Only 2.2% of participants in COAPT received full recommended doses of RAAS inhibitors, beta blockers and MRAs [19]. We found that TEER is safe and effective even in patients not receiving GDMT and interestingly their outcomes were similar to patients treated with GDMT. Theoretically the beneficial clinical and hemodynamic improvement following TEER might allow better tolerance for HF medications, for example reduction in diuretic doses after TEER might improve adherence to GDMT among HF patients with kidney dysfunction. This is supported by a recent study that demonstrated improved up titration of GDMT following TEER [20].

Several potential limitations of this study merit consideration. First, efforts to improve adherence of GDMT among patients with HFrEF are essential and our findings should not be interpreted as a recommendation to perform TEER prior to medical therapy optimization but rather not to exclude TEER in symptomatic patients that are unable to receive GDMT. Second, the similar outcomes between the study groups might be due to limited power of our study to detect the differences given the relatively small cohort of patients. Third, the present study is a retrospective analysis of collected data from 3 centers, and validation in a large multicenter prospective study is required to confirm these findings. Fourth, we did not include a control group receiving medical therapy only. Lastly, doses of medications other than furosemide were not captured in the database. The study was performed before SGLT2 inhibitors were introduced as standard therapy for CHF.

## Conclusions

Many patients with HFREF and FMR do not receive GDMT. Procedural success and one year mortality following TEER was similar in patients with and without GDMT. Lack of ability to receive GDMT should not preclude consideration of TEER for FMR in this significant population of patients.

## Data Availability

The corresponding author will provide data related to the manuscript on reasonable request.
